# Absence of *Vsx1* expression in the normal and damaged mouse cornea

**Published:** 2011-03-16

**Authors:** Tom Watson, Robert L. Chow

**Affiliations:** Department of Biology, University of Victoria, Victoria, BC V8W 3N5, Canada

## Abstract

**Purpose:**

To examine the expression of visual system homeobox 1 (*Vsx1*) in the mouse cornea and its potential role in the corneal wound response pathway.

**Methods:**

Expression of *Vsx1* was examined by quantitative reverse-transcription PCR (qRT–PCR) in corneal tissue from developing and adult mice and from mice that had undergone alkali-burn corneal wounding. Immunolabeling and *Vsx1* knock-in reporter gene expression in wild type and *Vsx1* null-mice were used to confirm the qRT–PCR data.

**Results:**

Using qRT–PCR, *Vsx1* expression was not detected in either the postnatal or adult mouse cornea or in corneas following wounding. This qRT–PCR data was supported by the absence of specific Vsx1 immunolabeling and *Vsx1* knock-in reporter expression in untreated and wounded corneas.

**Conclusions:**

In mice, *Vsx1* mRNA, protein or reporter gene expression is not detected in the normal or damaged cornea. These results make it uncertain what role *VSX1*/*Vsx1* plays in corneal biology. Future experiments examining the pathogenicity of *VSX1* mutations associated with corneal dystrophy are required to rule out species differences and possible non-cell autonomous roles for *VSX1* in the cornea.

## Introduction

Visual system homeobox 1 (*Vsx1*) is a paired-like homeobox gene, initially described in the goldfish retina [1] and later as *RINX* in the human retina [2]. In mice, *Vsx1* expression is first detected in the developing retinal inner nuclear layer (INL) cells at postnatal day 5 [3]. In the mature retina, *Vsx1* expression persists in the INL and is expressed in a subset ON and OFF cone bipolar cells [3,4]. *Vsx1* has been found to play a central role in bipolar cell differentiation. In *Vsx1* null mice bipolar cells are specified properly, but exhibit terminal differentiation defects characterized by reduced levels of bipolar marker expression in OFF cone bipolar cells [4,5]. Accompanying these defects in bipolar cell marker expression, *Vsx1* null mice have visual signaling defects that include a reduced electroretinogram b-wave and reduced OFF ganglion cell signaling, indicating a role for *Vsx1* in OFF bipolar cell function.

Despite the well characterized role for *Vsx1* in mouse retinal bipolar cells, the role of *VSX1*/*Vsx1* in human corneal disease is controversial. Eight dominant *VSX1* missense mutations associated with Posterior Polymorphous Corneal Dystrophy 1 (PPCD1; OMIM 122000) and keratoconus (OMIM 148300) have been identified [6-9]. Several of these mutations are compelling in that they alter highly conserved amino acid residues and/or are accompanied by electroretinogram defects indicative of retinal bipolar cell dysfunction as is observed in *Vsx1* null mice [4,5]. In contrast, other studies examining sporadic or familial keratoconus [10-13] and PPCD1 [14-16] have failed to reveal *VSX1* mutations. In addition, while *VSX1* corneal expression has been reported in adult [17] and neonatal [18] human cornea, other studies in mouse [3] and human [7,16,19] have failed to detect *VSX1/Vsx1* expression in the mature cornea. Interestingly, it has been reported that *VSX1* is upregulated in human and mouse corneal stromal keratocytes following corneal wounding [19]. This has led to the suggestion that *VSX1* functions as a corneal damage response gene and that corneal dystrophies associated with changes in *VSX1* are linked to defects in the corneal wound-healing pathway [19].

To investigate the role of *Vsx1* in the mouse cornea, we undertook a quantitative RT–PCR (qRT–PCR) approach to characterize the expression of *Vsx1* in the developing and mature mouse cornea. *Vsx1* expression was also examined in corneal tissue from mice that underwent alkali-burn corneal-wounding. qRT–PCR data on untreated and wounded corneas was complemented by immunohistological and *Vsx1* knock-in reporter gene expression studies. Using these approaches, we failed to detect *Vsx1* expression in both untreated and wounded corneas. Our findings make it uncertain what role, if any, *Vsx1* plays in the mouse cornea.

## Methods

### Animals

All experiments on wild type mice were performed on 129S1 inbred mice (The Jackson Laboratory, Bar Harbor, ME). *Vsx1:τLacZ* knock-in mice [4] were maintained on a 129S1 genetic background. All mouse work was done with approval from the University of Victoria Animal Care Committee, in accordance with the Canadian Council for Animal Care.

### RNA extraction

Mice were euthanized by cervical dislocation and tissues dissected in chilled phosphate buffered saline (PBS), transferred to 2 ml microcentrifuge tubes containing 1 ml Trizol (Invitrogen, Carlsbad, CA) and Precellys CK28 ceramic beads (Precellys, Montigny-le-Bretonneux, France) and then frozen on dry ice. Tissue homogenization was performed on a Precellys-24 homogenizer using two 10-s pulses. Homogenized tissue was spun for 10 min at 12,000× g at 2–8 °C, the supernatant transferred into fresh tubes and then kept at room temperature for 5 min. Next, 0.2 ml chloroform was added per ml of Trizol in the initial tubes, the tubes shaken, left to sit for 3 min at room temperature and then samples were centrifuged at 12,000× g for 15 min at 2–8 °C. The upper aqueous phase was then transferred into a fresh tube, 0.5ml isopropyl alcohol was added and samples were mixed by shaking. Following 10 min at room temperature and a 10-min centrifuge at 2–8 °C, 12,000× g, the supernatant was removed and resulting RNA pellet was washed using 75% ethanol before being stored in distilled RNase/DNase-free water (Gibco, Invitrogen,  Carlsbad, CA).

### qRT–PCR

Primers for *Vsx1* were designed using Primer 3Plus software while those for housekeeping gene glyceraldehyde-3-phosphate dehydrogenase (*Gapdh*), myofibroblast marker alpha-smooth muscle actin (*Acta2*) and rhodopsin (*Rho*) were taken from the literature (Table 1) [20-22]. Specific amplification was verified through comparison of PCR product sequences against published cDNA sequences. Cloning for sequencing was preformed using pGEMT Easy Vector protocol (Promega, Madison, WI) and QIAprep Miniprep kit (Qiagen Inc., Mississauga, Ontario). RT reactions were preformed as per Quantitect Reverse Transcription kit (Qiagen) protocol and reactions run as per Quantitect SYBR Green PCR kit (Qiagen): 15 min 95 °C, 35× (15 s 94 °C, 30 s 60 °C, 30 s 72 °C) using a Stratagene Mx300p (Agilent Technologies, Inc., Santa Clara, CA). Relative expression between samples was calculated using the 2^-ΔΔCt^ method [23] using the housekeeping gene *GAPDH* to normalize.

**Table 1:** Sequence and details of qRT–PCR primers.

**Gene**	**Primer**	**Sequence**	**Notes**
*Vsx1* primer pair 1	forward	TATGCCCCGTTTTAGTCTGG	both primers within exon 5; 143 bp product
	reverse	CCCAAAACAGACAACCCTTC	
*Vsx1* primer pair 2	forward	GGTTTTCACTGCCCATCAAC	reverse primer spans exon 3/4 junction; 109 bp product
	reverse	TTCCGGGAGCTCTGTTTTC	
*Vsx1* primer pair 3	forward	TGGGGATGCATAAAAAGTCC	forward primer spans 4/5 junction; 146 bp product
	reverse	GTTCTGGGTTGTTTCGTCTG	
*Gapdh*	forward	TGTGTCCGTCGTGGATCTGA	77 bp product [20]
	reverse	CCTGCTTCACCACCTTCTTGA	
*Rho* (rhodopsin)	forward	TTGCCACACTTGGAGGTGAA	forward primer spans exon 1/2 junction, 70 bp product [21]
	reverse	ACCACCACGTAGCGCTCAAT	
*Acta2* (alpha 2-SMA)	forward	ACTGGGACGACATGGAAAAG	240 bp product [22]
	reverse	CATCTCCAGAGTCCAGCACA	

### Corneal wounding procedure

Our alkali-burn corneal wounding protocol was based on previously published protocols [24,25]. Briefly, 4 μl of 0.2 M NaOH was applied to the corneas of anesthetized mice for 30 s followed by flushing with 10 ml of saline solution. Mice were then euthanized using cervical dislocation at desired time points post-wounding. Mice undergoing the wounding procedure were injected subcutaneously with buprenorphine (0.1 mg/kg) pre-wounding, and every 12 h for up to 3 days after the procedure to provide analgesia. One drop of Proparacaine was also applied to eyes immediately before NaOH application as a topical anesthesia. All mice were monitored throughout the experiments in accordance with University of Victoria animal care guidelines.

### Immunolabeling

Eyes removed from euthanized mice were transferred to chilled PBS and bisected longitudinally using scissors to expose inner ocular chambers. Bisected eyes were then fixed for 1 h in 4% paraformaldehyde/PBS on ice followed by washing in PBS and overnight cyroprotection in 30% sucrose/PBS at 4 °C. Tissue was frozen in Tissue-Tek OCT (Sakura Finetek, Torrance, CA) and sectioned at a thickness of 14 μm and stored at −20 °C. Sections were washed with PBS and cleared in 1% Triton X-100/PBS for 30 min. Blocking was performed in 10% horse serum/PBS for 1 h before incubating with primary rabbit anti-Vsx1 antibody [3] used at a 1:100 dilution in 1% horse serum/PBS. Secondary labeling was performed using an Alexa-Fluor-488 conjugated donkey anti-rabbit (1:100 in PBS). Nuclei were stained with Draq-5 (Biostatus, Leicestershire, UK). Slides were imaged using a Nikon confocal microscope (Nikon, Mississauga, Ontario). Post-imaging adjustment of levels in order to observe non-specific labeling was done in Adobe Photoshop (Adobe Systems Incorporated, San Jose, CA) by changing the maximum levels value from 256 to 26.

## Results

### Analysis of *Vsx1* mRNA expression in the postnatal and mature mouse cornea

**Figure 1:**
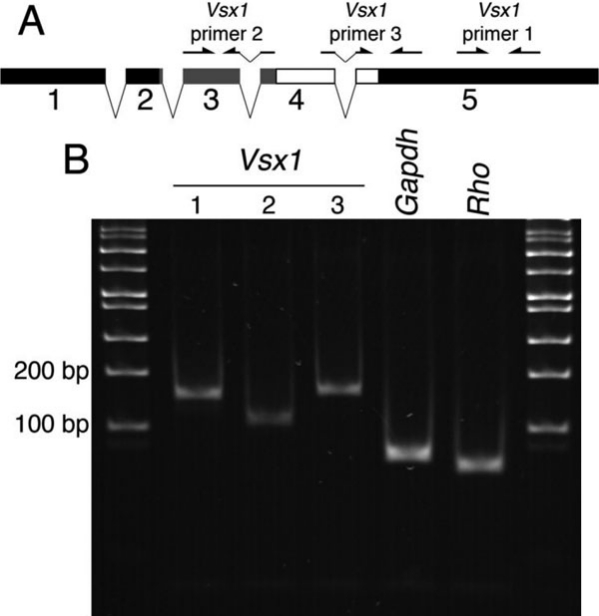
*Vsx1* qRT-PCR primer design. Locations of *Vsx1* qRT–PCR primers (see Table 1) on the *Vsx1* cDNA (**A**) and qRT–PCR samples of wild type mouse retina with *Vsx1* primers as well as *Gapdh* housekeeping and *Rho* sets (**B**) run on a 1.2% agarose gel. In mRNA schematic: Grey box – homeodomain, open box – CVC domain.

**Figure 2:**
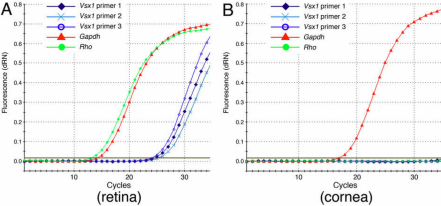
Representative *Vsx1* qRT-PCR amplification plots. Amplification plots of qRT–PCR of wild type mouse retinal (**A**) and corneal (**B**) samples using primers for *Vsx1*, *Gapdh* and *Rho* shown in Table 1. x-axis shows qRT–PCR cycle number, y-axis shows SYBR-green fluorescence values.

To examine *Vsx1* mRNA expression, 4 primer pairs spanning *Vsx1* exons 3–5 were designed. Only 3 of these primer pairs (Figure 1A) robustly amplified products from adult (2–8 months old) retinal cDNA of the predicted size (Figure 1B), with similar efficiencies (Figure 2A) and correct sequence (data not shown). In contrast to the retina, no qRT–PCR product was observed for any of the 3 *Vsx1* primer pairs when amplifying from adult corneal cDNA (Figure 2 B, n=3 mice). No specific qRT-PCT product was observed after up to 40 cycles (data not shown).

**Figure 3:**
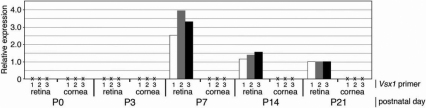
Expression of *Vsx1* in postnatal mouse retinal and corneal samples relative to P21 levels from qRT–PCR. Three animals were used for each time point. The y-axis shows *Vsx1* expression relative to expression on postnatal (P) day 21. x-axis shows postnatal day, tissue, and *Vsx1* primer set number.

We next examined retinal and corneal cDNA from wild type mice over the course of several postnatal time points (postnatal days 0, 3, 7, 14, and 21; n=3 mice for all time points; Figure 3). Consistent with the previously described onset of Vsx1 expression in mouse at postnatal day 5, retinal expression of *Vsx1* was detected in our samples at post-natal day 7 onward. In contrast, no *Vsx1* expression was detected in any of the corneal samples. Likewise, *Rho* (which served as a control for retinal contamination) was absent from the cornea.

### Analysis of *Vsx1* mRNA expression following corneal wounding

**Figure 4:**
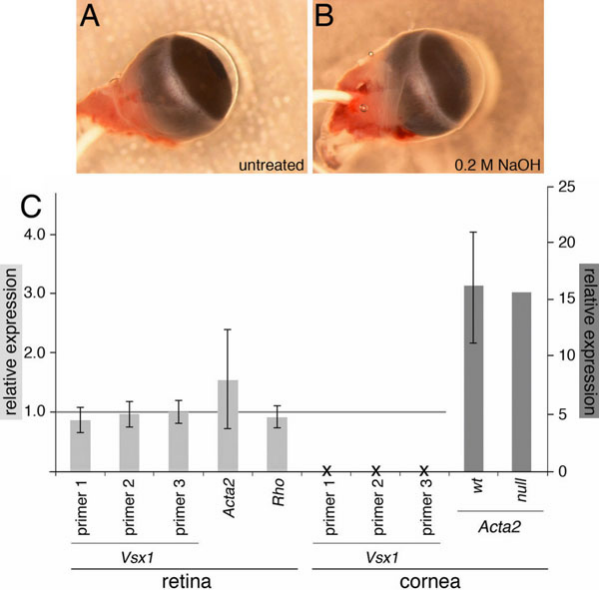
*Vsx1* qRT-PCR expression is not detected in the cornea following alkali wounding. orneal wounding was performed through application of 0.2 M NaOH, which resulted in significant structural changes after 3 days between untreated (**A**) and treated (**B**) mouse eyes. Relative expression of *Vsx1* in wounded versus unwounded eyes, normalized to *Gapdh*, from retinal and corneal samples is shown along with retinal *Rho* and *Acta2* expression. y-axis shows relative expression between wounded and untreated eyes while the x-axis indicates primer set (*Vsx1* sets 1–3, *Acta2*, and *Rho*) and tissue (retinal or corneal).

Corneal wounding experiments were performed on 8 wild type mice with one eye receiving an alkali burn and the other serving as an untreated control (4 mice with treated left eye, 4 with treated right). Wounded corneas appeared cloudy from 1 day post-wounding. Retinal and corneal tissue was taken 3 days after the corneal wounding with NaOH and the wounded morphology of treated corneas was confirmed through examination during dissection (Figure 4A,B). Alkali treated corneas were no longer transparent, appeared thicker and had considerably pitted surfaces compared to the unwounded control eyes from the same mouse. qRT–PCR results found no significant change in *Vsx1* expression post-wounding in the retina samples. *Vsx1* expression was not observed in untreated or alkali-wounded corneal samples but *Acta2*, a gene that is known to be upregulated following corneal alkali burn [25] was significantly unregulated by an average of 16.3 times in treated relative to untreated control eyes for both wild type and *Vsx1^τLacZ/τLacZ^* mice (Figure 4C). A time course experiment set-up identically to the 3 day wounding found no *Vsx1* expression in the corneas of wild type mice 6 h, 1 day, 3 days, 7 days or 14 days post-wounding although *Acta2* was upregulated in each case (data not shown).

### Histological examination of *Vsx1* expression following corneal wounding

**Figure 5:**
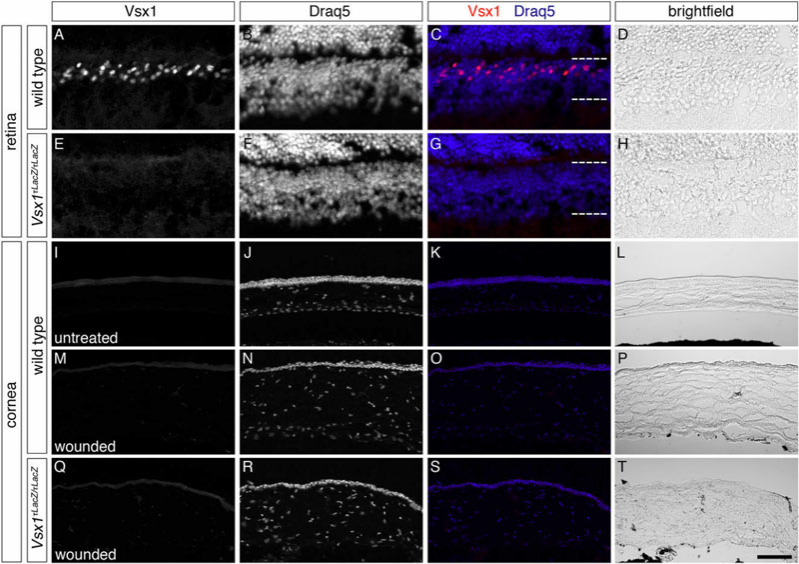
Vsx1 immunolabeling is not detected in normal or alkali-wounded corneas. sx1 immunolabeling (**A**, **E**, **I**, **M**, **Q**) of adult mouse retina (**A**-**H**) and cornea (**I**-**T**). Vsx1 immunolabeling of bipolar cells is present in wild type retinal section (**A**-**D**) but not in *Vsx1^τLacZ/τLacZ^* mice (**E**-**H**). Corneal sections from untreated mice (**I**-**L**) and from corneas receiving alkali-burn (**M**-**T**) were imaged for Vsx1 immunolabeling using the same imaging settings as those used for imaging retina. Sections were co-labeled with Draq5 nuclear stain.

**Figure 6:**
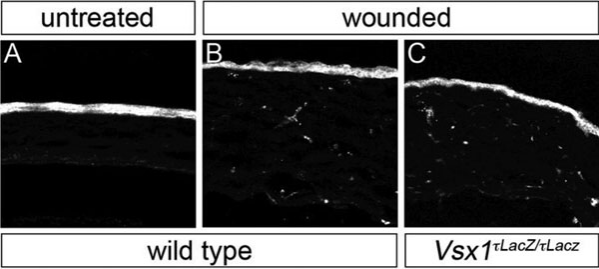
Non-specific immunolabeling of alkali-wounded corneas. Panels **A**-**C** correspond to panels **I**, **M**, and **Q**, respectively, from Figure 5. The image pixel intensity levels have been elevated by an order of magnitude.

**Figure 7:**
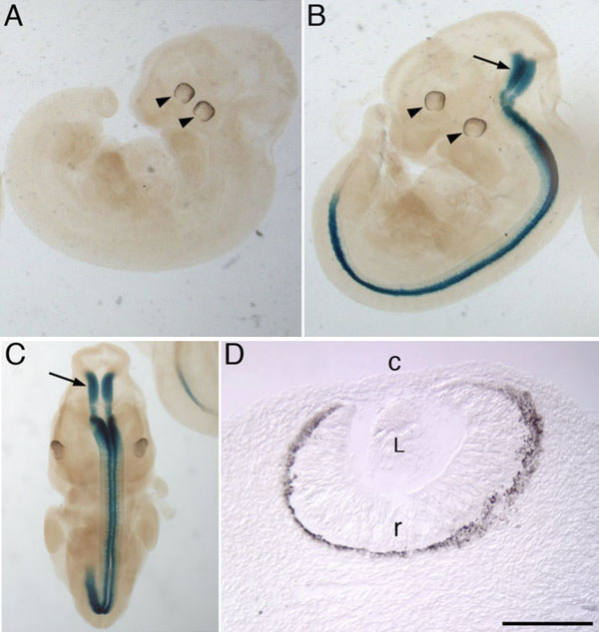
*Vsx1* knock-in reporter gene expression at embryonic day 11.5. X-gal staining of wild type (**A**) and *Vsx1^τLacZ/τLacZ^* (**B**-**D**) mouse embryos embryonic day 11.5. Xgal staining is detected in the developing hindbrain (arrows in **B** and **C**) and spinal cord but is not detected in the developing eye (**A**, **B** - arrowhead). **D**: Cross-section through the eye of the embryo from panel **B** shows that ocular expression is undetectable in the developing retina (r), lens (L) and corneal region (c). Scale bar in **D**=50 μm.

**Figure 8:**
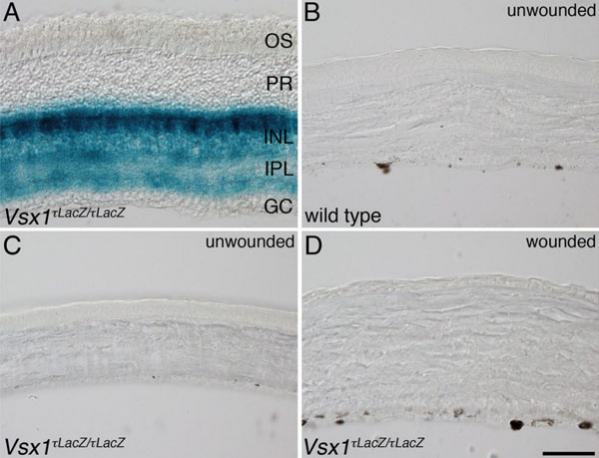
*Vsx1* knock-in reporter gene expression is not detected in normal or alkali-wounded corneas. X-gal staining of retina (**A**), untreated cornea (**C**) and alkali-burn damaged cornea (**D**) from *Vsx1^τLacZ/τLacZ^* mice as well as the untreated cornea from a wild type mouse (**B**).

Immunohistological analysis aimed to confirm the qRT–PCR results in the cornea. No specific Vsx1 immunolabeling was observed in the corneas of alkali-burn wounded or untreated mice as compared to *Vsx1^τLacZ/τLacZ^* mice, which do not have Vsx1-immunolabeling [4] and therefore serve as a negative control (Figure 5). It was noted that when the post-acquisition imaging pixel intensity level was elevated by an order of magnitude higher than the optimal near-saturation levels used for imaging retinal Vsx1, non-specific labeling was observed in both wild type and *Vsx1^τLacZ/τLacZ^* alkali-burn treated corneas but not in untreated corneas (Figure 6). We next examined β-galactosidase knock-in reporter expression in normal and wounded corneas of *Vsx1^τLacZ/τLacZ^* mice. The *Vsx1 τLacZ* knock-in reporter recapitulates the normal *Vsx1* expression pattern in retinal bipolar cells [4] and, although it is present in the developing ventral hindbrain and spinal cord, it is not detected from the embryonic retina and cornea (Figure 7 and data not shown). Similar to the non-specific Vsx1 immunolabeling observed in the wounded cornea, a high degree of non-specific β-galactosidase immunolabeling was also observed in wild type and wounded cornea (data not shown). We therefore examined β-galactosidase activity using the X-gal (5-bromo-4-chloro-3-indolyl-beta-D-galacto-pyranoside) chromogenic substrate. In contrast to robust β-galactosidase activity in the *Vsx1^τLacZ/τLacZ^* retina, no activity was observed in wounded or untreated corneas (Figure 8).

## Discussion

The role of *VSX1*/*Vsx1* in the corneal dystrophies keratoconus and posterior polymorphism dystrophy (PPCD1) and, the issue of whether it is expressed in the cornea has been a subject of considerable controversy. This study aimed to resolve the question of corneal *Vsx1* expression in mice and evaluate a possible explanation for the association of *VSX1* mutations with corneal disease.

For qRT–PCR, the use of three different Vsx1 primers spaced over three of the five exons of the *Vsx1* gene, two of which were intron-spanning, aimed to ensure reliable detection and reproducibility of any *Vsx1* mRNA expression in samples. Further confidence in these primers was gained through comparison to previous *Vsx1* expression studies. For example, in this study, postnatal *Vsx1* expression in the mouse retina was only detected after postnatal day 3, which is consistent with *Vsx1* expression detected by in situ hybridization at post-natal day 5 [3]. In contrast to the very low levels of *VSX1* neonatal expression reported between day of birth and 9 months of age, we did not observe any *Vsx1* corneal expression in the postnatal mouse at stages before and after eye opening. These findings suggest possible differences in the role of *VSX1/Vsx1* in human and mouse corneal biology.

Our inability to detect any corneal Vsx1 expression in adult wild-type mouse corneal tissue is consistent with the results of previous studies [3,7,26] that have failed to detect *Vsx1* corneal expression. We thus investigated whether *Vsx1* might be expressed as part of the wound healing response in the cornea as previously proposed [19]. The significant upregulation of *Acta2*, signifying the keratocyte to myofibroblast transformation associated with wound response in the cornea, in addition to the morphology changes seen in our wounded corneal samples both indicate that the alkali insult was successful in stimulating the corneal wounding response. Despite this response, qRT–PCR, immunolabeling and reporter gene expression revealed no specific corneal *Vsx1/*Vsx1 expression in untreated or alkali-burn damaged corneas at time points ranging from 6 h to 14 days post-wounding. Interestingly, there did appear to be a slight increase in non-specific labeling in alkali-burn damaged corneas that was not observed in untreated corneas. However, since this labeling was also present in *Vsx1^τLacZ/τLacZ^* mice, which lack Vsx1 immunolabeling, this suggests that wounded corneas are more prone to non-specific labeling than untreated corneas. Furthermore, following alkali-burn, no obvious changes in the corneal wound response at the histological level or in the upregulation of *Acta2* were observed in *Vsx1^τLacZ/τLacZ^* mice, indicating that *Vsx1* is not essential for these features of the wound response. Our findings call into question the potential association of *Vsx1* with the wound response pathway in the mouse cornea.

In this study, we show that *Vsx1* is not expressed at detectable levels in the cornea at all stages examined. We demonstrated that *Vsx1* is not detected in the cornea following alkali-burn damage using qRT–PCR, immunohistology and *Vsx1* knock-in reporter expression. It remains possible that *VSX1/Vsx1* contributes in corneal pathology through a cell non-autonomous mechanism, however this possibility has yet not been investigated. Our data suggests that in addition to the genetic heterogeneity and complexity that has been predicted for keratoconus and PPCD1, there may also be species differences in role of *VSX1/Vsx1* in human and mouse corneal biology.

## References

[1] Levine EM, Hitchcock PF, Glasgow E, Schechter N (1994). Restricted expression of a new paired-class homeobox gene in normal and regenerating adult goldfish retina.. J Comp Neurol.

[2] Hayashi T, Huang J, Deeb SS (2000). RINX(VSX1), a novel homeobox gene expressed in the inner nuclear layer of the adult retina.. Genomics.

[3] Chow RL, Snow B, Novak J, Looser J, Freund C, Vidgen D, Ploder L, McInnes RR (2001). Vsx1, a rapidly evolving paired-like homeobox gene expressed in cone bipolar cells.. Mech Dev.

[4] Chow RL, Volgyi B, Szilard RK, Ng D, McKerlie C, Bloomfield SA, Birch DG, McInnes RR (2004). Control of late off-center cone bipolar cell differentiation and visual signaling by the homeobox gene Vsx1.. Proc Natl Acad Sci USA.

[5] Ohtoshi A, Wang SW, Maeda H, Saszik SM, Frishman LJ, Klein WH, Behringer RR (2004). Regulation of retinal cone bipolar cell differentiation and photopic vision by the CVC homeobox gene Vsx1.. Curr Biol.

[6] Bisceglia L, Ciaschetti M, De Bonis P, Campo PA, Pizzicoli C, Scala C, Grifa M, Ciavarella P, Delle Noci N, Varia F, Macaluso C, Zelante L (2005). VSX1 mutational analysis in a series of Italian patients affected by keratoconus: detection of a novel mutation.. Invest Ophthalmol Vis Sci.

[7] Héon E, Greenberg A, Kopp KK, Rootman D, Vincent AL, Billingsley G, Priston M, Dorval KM, Chow RL, McInnes RR, Heathcote G, Westall C, Sutphin JE, Semina E, Bremner R (2002). Stone EM. VSX1: a gene for posterior polymorphous dystrophy and keratoconus.. Hum Mol Genet.

[8] Mintz-Hittner HA, Semina EV, Frishman LJ, Prager TC, Murray JC (2004). VSX1 (RINX) mutation with craniofacial anomalies, empty sella, corneal endothelial changes, and abnormal retinal and auditory bipolar cells.. Ophthalmology.

[9] Valleix S, Nedelec B, Rigaudiere F, Dighiero P, Pouliquen Y, Renard G, Le Gargasson JF, Delpech M (2006). H244R VSX1 is associated with selective cone ON bipolar cell dysfunction and macular degeneration in a PPCD family.. Invest Ophthalmol Vis Sci.

[10] Aldave AJ, Yellore VS, Salem AK, Yoo GL, Rayner SA, Yang H, Tang GY, Piconell Y, Rabinowitz YS (2006). No VSX1 gene mutations associated with keratoconus.. Invest Ophthalmol Vis Sci.

[11] Liskova P, Ebenezer ND, Hysi PG, Gwilliam R, El-Ashry MF, Moodaley LC, Hau S, Twa M, Tuft SJ, Bhatacharya SS (2007). Molecular analysis of the VSX1 gene in familial keratoconus.. Mol Vis.

[12] Stabuc-Silih M, Strazisar M, Hawlina M, Glavac D (2010). Absence of pathogenic mutations in VSX1 and SOD1 genes in patients with keratoconus.. Cornea.

[13] Tang YG, Picornell Y, Su X, Li X, Yang H, Rabinowitz YS (2008). Three VSX1 gene mutations, L159M, R166W, and H244R, are not associated with keratoconus.. Cornea.

[14] Aldave AJ, Yellore VS, Principe AH, Abedi G, Merrill K, Chalukya M, Small KW, Udar N (2005). Candidate gene screening for posterior polymorphous dystrophy.. Cornea.

[15] Gwilliam R, Liskova P, Filipec M, Kmoch S, Jirsova K, Huckle EJ, Stables CL, Bhattacharya SS, Hardcastle AJ, Deloukas P, Ebenezer ND (2005). Posterior polymorphous corneal dystrophy in Czech families maps to chromosome 20 and excludes the VSX1 gene.. Invest Ophthalmol Vis Sci.

[16] Hosseini SM, Herd S, Vincent AL, Heon E (2008). Genetic analysis of chromosome 20-related posterior polymorphous corneal dystrophy: genetic heterogeneity and exclusion of three candidate genes.. Mol Vis.

[17] Semina EV, Mintz-Hittner HA, Murray JC (2000). Isolation and characterization of a novel human paired-like homeodomain- containing transcription factor gene, VSX1, expressed in ocular tissues.. Genomics.

[18] Houweling AC, Dildrop R, Peters T, Mummenhoff J, Moorman AF, Ruther U, Christoffels VM (2001). Gene and cluster-specific expression of the Iroquois family members during mouse development.. Mech Dev.

[19] Barbaro V, Di Iorio E, Ferrari S, Bisceglia L, Ruzza A, De Luca M, Pellegrini G (2006). Expression of VSX1 in Human Corneal Keratocytes during Differentiation into Myofibroblasts in Response to Wound Healing.. Invest Ophthalmol Vis Sci.

[20] Baron V, De Gregorio G, Krones-Herzig A, Virolle T, Calogero A, Urcis R, Mercola D (2003). Inhibition of Egr-1 expression reverses transformation of prostate cancer cells in vitro and in vivo.. Oncogene.

[21] Brafman A, Mett I, Shafir M, Gottlieb H, Damari G, Gozlan-Kelner S, Vishnevskia-Dai V, Skaliter R, Einat P, Faerman A, Feinstein E, Shoshani T (2004). Inhibition of oxygen-induced retinopathy in RTP801-deficient mice.. Invest Ophthalmol Vis Sci.

[22] Fu P, Liu F, Su S, Wang W, Huang XR, Entman ML, Schwartz RJ, Wei L, Lan HY (2006). Signaling mechanism of renal fibrosis in unilateral ureteral obstructive kidney disease in ROCK1 knockout mice.. J Am Soc Nephrol.

[23] Livak KJ, Schmittgen TD (2001). Analysis of relative gene expression data using real-time quantitative PCR and the 2(-Delta Delta C(T)). Methods..

[24] Gönül B, Erdogan D, Ozogul C, Koz M, Babul A, Celebi N (1995). Effect of EGF dosage forms on alkali burned corneal wound healing of mice.. Burns.

[25] Lee SH, Leem HS, Jeong SM, Lee K (2009). Bevacizumab accelerates corneal wound healing by inhibiting TGF-beta2 expression in alkali-burned mouse cornea.. BMB Rep.

[26] Hayashi T, Huang J, Deeb SS (2005). Expression of rinx/vsx1 during postnatal eye development in cone-bipolar, differentiating ganglion, and lens fiber cells.. Jpn J Ophthalmol.

